# Multifaceted role of H_2_O_2_ in the solvothermal synthesis of green-emitting nitrogen-doped graphene quantum dots†

**DOI:** 10.1039/d4sc07896a

**Published:** 2025-01-28

**Authors:** Clara Carrera, Alejandro Galán-González, Wolfgang K. Maser, Ana M. Benito

**Affiliations:** a Instituto de Carboquímica (ICB-CSIC) C/Miguel Luesma Castán 4 E-50018 Zaragoza Spain abenito@icb.csic.es

## Abstract

Fluorescent nitrogen-doped carbon dots (N-GQDs) with long-wavelength emission properties are of increased interest for technological applications. They are widely synthesized through the solvothermal treatment of graphene oxide (GO) using *N*,*N*-dimethylformamide (DMF) as a cleaving and doping agent. However, this process simultaneously generates undesired interfering blue-emissive by-products. In this study, we present a straightforward method for synthesizing N-GQDs exclusively exhibiting green fluorescence. The key innovation lies in the addition of hydrogen peroxide (H_2_O_2_) to the DMF-driven one-pot solvothermal cleavage process. Systematically controlling the reaction conditions, we elucidate the threefold beneficial role of H_2_O_2_: first, it acts as a radical source facilitating the degradation of DMF and the generation of nitrogen-containing radicals, essential for N-GQD formation; second, it prevents the thermal reduction of GO, thus ensuring persistent reaction pathways with DMF-derived radicals; and third, it suppresses the self-reaction of DMF-derived radicals, thereby avoiding the formation of undesired blue-fluorescent by-products. Our findings on the reaction mechanism and the advantageous role of H_2_O_2_ open new possibilities for the rational design of N-GQDs genuinely emitting at long wavelengths.

## Abstract 2

Solvothermal synthesis of N-GQD using H_2_O_2_ in a DMF-driven process, ensuring green emission, suppressing blue by-products, and enabling precise control of long-wavelength fluorescence for advanced applications.

## Graphical abstract

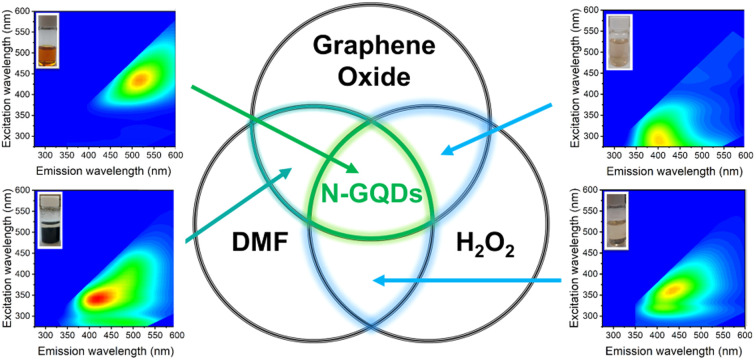

## 1 Introduction

Graphene Quantum Dots (GQDs) have gathered great attention in recent years due to their outstanding photoluminescence combined with interesting properties such as water solubility, stability, non-toxicity and good biocompatibility. These characteristics confer them applicability in multiple fields such as bioimaging, sensing, photocatalysis, optoelectronics or energy.^1–6^

GQDs can be synthesized by top-down or bottom-up approaches.^7–9^ Among the top-down strategies, the solvothermal treatment of carbon nanomaterials stands out due to its ability to fine tune the properties of these GQDs. This method allows the use of different solvents or reagents to modify the final structure of the resulting GQDs. By carefully designing the experimental conditions, key properties such as particle size, functional groups or heteroatom doping can be tailored.^10–15^ This process is based on the fragmentation of graphitic-like precursor structures to form GQDs. Graphene oxide (GO) is frequently chosen as starting carbon nanomaterial due to the abundant presence of oxygen functional groups (OFGs).^16,17^ During solvothermal processes, the OFGs facilitate the fragmentation of GO and the subsequent formation of GQDs. This fragmentation is usually carried out by cleaving agents, such as acids or oxidants that increase the reactivity of GO, which is thermally reduced during the thermal treatment. Among them, hydrogen peroxide (H_2_O_2_) is commonly employed in the cleavage process of GO to produce blue-emitting fluorescent GQDs.^18^

As long-wavelength emitting GQDs broaden the application portfolio, current research efforts focus on the synthesis of GQDs with red-shifted emission characteristics.^19–22^ Heteroatom-doping strategies incorporating nitrogen, boron, sulphur or phosphor in the GQD structure are frequently employed to modify the fluorescence behaviour.^23–25^ In the case of nitrogen-doped GQDs (N-GQDs), the specific type of functional group containing the nitrogen atom is key to achieve fluorescence emission at long-wavelengths.^26^ As such, an increasing amount of pyridinic-N is known to increase the intensity of the fluorescent emission^27^ while the presence of graphitic-N is reported to produce a redshift on the emission wavelength.^28^

A common method to produce N-GQDs involves the solvothermal treatment of GO in *N*,*N*-dimethylformamide (DMF), which acts as both the solvent and the nitrogen source.^29,30^ The reaction pathway of this method is thought to be directly influenced by the DMF instability under the treatment conditions. DMF is reported to decompose at high temperature to ultimately form carbon monoxide and dimethylamine. This amine can then react with the OFGs of GO to form green emitting N-GQDs.^30^ However, the targeted green fluorescence of the resulting N-GQDs is typically compromised by the contribution of an undesired blue emission. Although its appearance is not explicitly addressed in the literature, understanding its origin is of critical importance for suppressing this interfering component and improving the synthesis pathways of long-wavelength fluorescent N-GQDs.

In this work, we employ a straightforward one-pot solvothermal method, which profits from the unexplored synergetic combination of DMF with H_2_O_2_ to produce N-GQDs exhibiting exclusively green fluorescence emission. Furthermore, the influence of each component in the process is studied in detail to identify the origin of the blue fluorescence contribution typically observed in the thermal treatment using DMF. Likewise, we investigate the formation of fluorescent by-products derived solely from the solvent degradation. This approach provides essential insights into the unique role of H_2_O_2_ facilitating the efficient synthesis of long-wavelength fluorescent N-GQDs and preventing the interference from undesired blue-fluorescent DMF-derived by-products.

## 2 Experimental

### 2.1. Materials

Graphite flakes were supplied by Sigma Aldrich while *N*,*N*-dimethylformamide and KMnO_4_ were purchased from Fisher Scientific. H_2_SO_4_ (98%), HCl (37%), NaNO_3_ and H_2_O_2_ 30% (v/v) were provided by Labkem (Barcelona, Spain). The 0.45 μm PTFE hydrophilic filters used for the N-GQDs purification were bought from CHMLAB Group (Barcelona, Spain).

### 2.2. Synthesis

As a first step, graphene oxide was synthetized from graphite using Hummers' modified method (ESI, Section S1, and Fig. S1†).^31,32^ In a typical synthesis of N-GQDs, 20 mg of GO were dispersed in 9 mL of DMF using an ultrasound bath during 30 minutes. Thereafter, 1 mL of H_2_O_2_ (30%) was added and stirred during 3 minutes. The mixture was then transferred to a 40 mL Teflon-lined autoclave and heated to 200 °C during 5 hours (defined as standard conditions). Once the reaction was completed, the autoclave was cooled down naturally to room temperature. The obtained dispersion was filtered using a 0.45 μm PTFE filter to remove the larger particles and unreacted GO. As a final step, the solvent was rotary evaporated to obtain the N-GQDs. All reactions were carried out by using a total volume of 10 mL of solvent. Correspondingly, reactions without H_2_O_2_ were performed using 10 mL of DMF. For those carried out in absence of DMF, the solvent was replaced by 9 mL of distilled water. Likewise, on the reactions in absence of GO the sonication step was omitted.

### 2.3. Characterisation

The photoluminescent properties of the previously filtered solutions were determined by using a 10 mm path-length quartz cuvette. The fluorescence spectra were measured on a Horiba Jobin Yvon Fluoromax-P and UV-vis absorption spectra were analysed with a Shimadzu UV-2401 PC spectrophotometer. The photoluminescence of the samples can be compared since all experiments were performed employing 10 mL of total volume. Thus, the differences among them can be attributed to concentration changes or to the formation of new species. Compositional analysis of GO, N-GQDs and DMF by-products (drop-casted onto a Si foil) was performed using X-ray photoelectron spectroscopy (XPS) on an ESCAPlus Omicron XPS with Mg K_α_ radiation (1253.6 eV, 300 W). Transmission electron microscopy (TEM) images were acquired using a FEI Tecnai F30 microscope operating at 200 kV. X-ray diffraction (XRD) patterns of the filtered solids were obtained using a Bruker D-8 Advance diffractometer with Cu K_α_ radiation (*λ* = 1.541 Å) in the 2*θ* range from 5° to 80°. Thermogravimetric analysis (TGA) was performed in a SETSYSTS EVOLUTION thermobalance (Setaram) under nitrogen atmosphere using a ramp of 5 °C min^−1^ from 20 °C to 800 °C. Fourier-transform infrared (FT-IR) spectra were acquired in a Vertex 70 apparatus (Bruker), using attenuated total reflectance (ATR) from N-QGDs deposited on a KBR foil. Raman spectra were obtained from a Micro-Raman Horiba Jobin Yvon HR800 UV spectrometer with CCD detector using an excitation wavelength of 532 nm probing N-QGDs deposited on the KBR foil.

## 3 Results and discussion

**Fig. 1:**
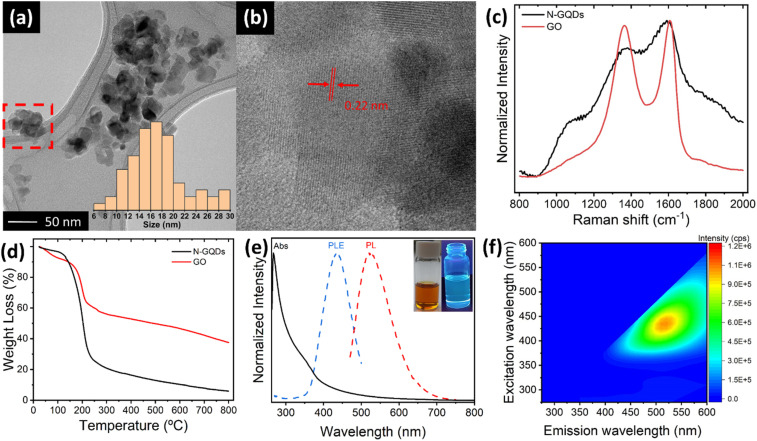
(a) TEM image and histogram (inset) of agglomerated N-GQDs. (b) Magnified TEM image of the red-marked zone in (a), indicating the presence of crystalline lattice fringes within agglomerated N-GQDs. (c) Raman spectra of N-GQDs and GO. (d) TGA of N-GQDs and GO. (e) Normalized UV-vis, photoluminescence excitation (PLE) and photoluminescence emission (PL) spectra of the N-GQD solution in DMF acquired at maximum emission and excitation intensities at 525 nm and 430 nm, respectively. Inset shows the N-GQD solution under day-light and its fluorescence under UV-light. (f) 2D photoluminescence map of N-GQDs solution in DMF.

**Fig. 2:**
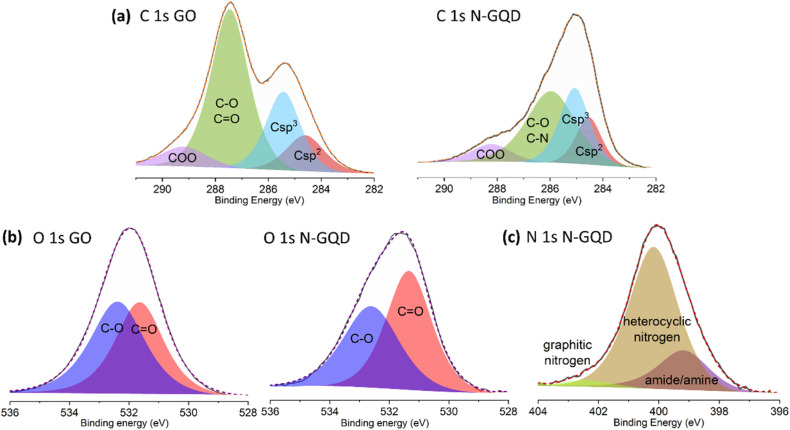
High resolution XPS spectra of GO and N-GQDs: (a) C 1s orbitals; (b) O 1s orbitals and (c) N 1s orbitals.

N-doped GQDs were prepared through a solvothermal treatment of GO with DMF and H_2_O_2_ at 200 °C in an autoclave for a time of 5 hours. After the reaction, two distinct fractions were obtained: a black solid precipitate composed of GO sheets and a bright orange solution. The precipitate, analysed in detail by TEM studies (ESI, Fig. S1†), consists of restacked GO sheets presenting very rough and heavily attacked edge structures, suggesting that N-GQDs most likely are cleaved from the edge sites of GO sheets. The solution fraction reveals the formation of N-GQDs. TEM images (Fig. 1a, b and ESI, S2†) show the presence of aggregated particles of N-GQDs with mean sizes of 16 nm (see histogram in inset of Fig. 1a), in-line with observations by other authors.^33–35^ Crystalline lattice fringes in different parts of the aggregates are identified, exhibiting a size of about 0.22 nm (Fig. 1b). Being in agreement with the typical in-plane (100) lattice distance of a graphene sheet, this observation underscores the graphemic structure of the N-GBDs, and thus the direct relationship with the parent GO sheets. Although the determination of the crystalline domain sizes is hampered by the aggregate structure exhibiting fringes from overlapping N-GQDs, they do not exceed values of 10 nm. This falls in the range of domain sizes enabling quantum-size effects,^36^ thus corroborating the creation of N-GQDs from GO sheets. The normalized Raman spectra of N-GQDs and the parent GO (Fig. 1c) exhibit the typical G and D bands at 1605 cm^−1^ and 1366 cm^−1^, characteristic for the presence of both sp^2^ carbon and sp^3^ hybridized carbon domains, respectively,^37^ further underscoring the direct relationship between GO and N-GQDs. Although the apparent change in the intensity ratio of the D and G band their increased bandwidth for N-GQDs is probably influenced by the strong fluorescent background it confirms the successful transformation of the non-fluorescent parent GO sheets into fluorescent N-GQDs of smaller crystallite domain sizes. TGA studies (Fig. 1d, and ESI, Section S4†) reveal an accelerated decomposition underscoring the lower thermal stability of N-QGCDs compared to GO, as a consequence of their increased surface area, as well as their higher density of functional groups per mass unit, in agreement with FT-IR studies (ESI, Section S4†). The UV-vis absorption spectrum of the N-GQD solution in DMF (Fig. 1e) displays a peak at about 270 nm accompanied by a broad shoulder extending from about 320 nm to 380 nm, followed by an absorption tail. The first peak is attributed to π–π* transitions of C=C bonds of sp^2^ domains, while the second is associated to sp^3^ n–π* transitions associated to C=O and conjugated C–N/C=N bonds within the N-GQDs.^38^ The low intensity absorption tail is either caused by the presence of dense intra-gap-states comprising edge states, nitrogen states and oxygen surface states^39^ or by scattering effects from aggregated N-GQDs.^39–41^ Additionally, Fig. 1e depicts the normalized photoluminescence excitation (PLE) and emission (PL) spectra of N-GQDs in DMF, acquired at maximum emission and excitation intensities at 525 nm and 430 nm, respectively. Since the PLE spectrum entirely falls in the region governed by the absorption tail, the photoluminescence of N-GQDs most likely involves both, densely lying intra-gap states comprising edge states, nitrogen states and oxygen surface states,^35,39^ or aggregation induced energy states, significantly enhanced in DMF.^39–41^ The 2D photoluminescence map (Fig. 2f) clearly confirms that N-GQDs only exhibit an emission for excitation wavelengths higher than 370 nm. Furthermore, the 2D map reveals an excitation-dependent emission behaviour, similar to other types of GQDs described in the literature.^35,39,41^

To demonstrate the successful cleavage of GO and the incorporation of nitrogen into the GQDs structure, XPS measurements of drop-casted samples were carried out. Analyses of the XPS survey spectra (ESI, Fig. S4 and Table S1†) show for pristine GO a composition of 65 at% carbon and 35 at% oxygen. By contrast, the N-GQDs reveals a composition of 76 at% carbon and 13 at% oxygen, while the remaining 11.0 at% account for the incorporation of nitrogen. The decrease in oxygen content is attributed to the cleaving process that GO undergoes during the thermal treatment.^29^ Interestingly, deconvolution of the high resolution C 1s XPS spectra (Fig. 2a) discloses a significant reduction in the contribution of signals corresponding to single (C–O) and double (C=O) carbon–oxygen bonds in N-GQDs compared to pristine GO. The signal related to these groups, initially at 287.4 eV in GO, shifts to 286.0 eV in N-GQDs and displays a notable decrease in intensity, reducing its contribution from 54.2 at% to 45.1 at%. Importantly, this signal also includes the contribution from the newly formed C–N bonds, potentially contributing to the underestimation of the overall reduction of the C–O and C=O OFG content. Additionally, the contribution of the two peaks associated with C sp^2^ and C sp^3^ bonds (around 284 eV and 285 eV, respectively) is more pronounced in N-GQDs (16.4 at% and 29.6 at%) compared to GO (13.1 at% and 24.9 at%), in agreement with the cleaving process of GO and the simultaneous removal of OFGs.

High resolution O 1s XPS spectra of GO and N-GQDs (Fig. 2b) provide information on the nature of the oxygen functional groups. Both spectra were deconvoluted into two components, assigned to C–O (epoxy, alcohols) at 532.5 eV and C=O (mainly carboxylic groups) at 531.5 eV. In GO, the contributions of both C–O and C=O signals are similar, with C–O being slightly more significant. However, this trend changes after the cleaving process, leading to a reduction of the C–O component from 52.8 at%, for GO, to 45.7 at%, for N-GQDs (ESI, Table S2†). This decrease in the amount of C–O functional groups confirms the preferential cleavage of GO sheets through the more reactive C–O groups (epoxy, alcohols), as suggested in studies involving their reaction with H_2_O_2_ (ref. 18) or DMF.^30^

Deconvolution of the high resolution N 1s XPS spectrum of the N-GQDs reveals the presence of three types of nitrogen species (Fig. 1c). The signal at 399.2 eV corresponds to amides or amines derived from the decomposition of DMF. The main signal at 400.2 eV (74.8 at%) is related to C–N bonds within a pyrrolic or pyridinic conjugated environment, providing evidence for the formation of these specific nitrogenated heterocycles. Interestingly, a minor contribution is observed for graphitic nitrogen. The observed redshift in the green fluorescence of N-GQDs implies the presence of a minor amount of graphitic nitrogen, which is known to influence the final emission wavelength of these materials.^28^ The existence of nitrogen-containing functional groups including graphitic nitrogen is further confirmed by FT-IR results (see ESI, Section 4†).

**Fig. 3:**
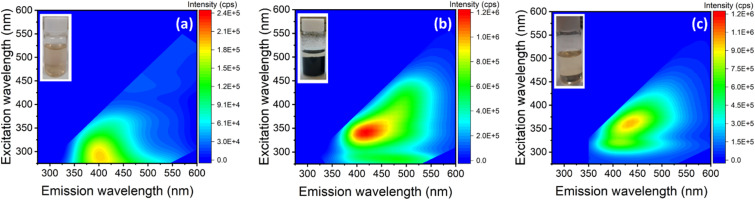
2D photoluminescence maps of the solution obtained by the reaction of (a) GO and H_2_O_2_, (b) GO and DMF, and (c) DMF alone. (Inset) Photo of the solutions under day-light.

Next, the influence of each individual reactant separately (DMF and H_2_O_2_) in our synthetic approach is put under close scrutiny. The reaction of GO and H_2_O_2_ leads to a slightly pale-brown coloured solution without any residual solid precipitate (inset of Fig. 3a), as opposed to the situation when all three reactants are jointly employed (inset of Fig. 1a). The 2D photoluminescence map (Fig. 3a) of the GO-H_2_O_2_ solution shows a remarkable decrease in the overall photoluminescence intensity and a significant shift of the maximum photoluminescence emission centre down to 400 nm, obtained at a maximum excitation wavelength at 310 nm. Since the photoluminescence of GQDs is highly size-dependent,^42,43^ the decreased intensity and observed blueshift in the H_2_O_2_-only treatment suggest an overly aggressive reaction process. This likely leads to excessive oxidation of GO, resulting in the formation of smaller nanoparticles exhibiting a new low intensity and low PLE fluorescent center, in contrast to the N-GQDs.

The solvothermal treatment of 20 mg GO in DMF in the absence of H_2_O_2_ leads to a pale-yellow solution and a black, spongy solid (Inset of Fig. 3b). The 2D photoluminescence map (Fig. 3b) of the filtered solution shows two PL emission centres, with maxima located at 415 nm and 495 nm upon excitation at 340 and 410 nm, respectively. The presence of these two PL centres evidences the existence of two distinct fluorescence processes. Most likely, the green contribution is intrinsic to the N-GQDs formed upon GO cleavage, as their emission properties resemble those of the product obtained using all three components (Fig. 1a). Additionally, XRD analysis of the solid fraction confirms the reduction of GO during the solvothermal process, explaining the non-fluorescent character of the precipitated product (ESI, Section S6†). Thus, the combination of both H_2_O_2_ and DMF is key to obtain N-GQDs. The oxidative nature of H_2_O_2_ partially hinders the characteristic thermal reduction of GO, while DMF inhibits the overoxidation that occurs when only H_2_O_2_ is used in the reaction.

Further investigation into the GO cleaving process with DMF, using two different amounts of GO (50 mg and 3.5 mg), confirms that the green emission peak is directly related to the presence of GO (ESI, Fig. S6†), since its intensity varies with the concentration of GO. By contrast, the unaltered blue emission (415–450 nm) in all spectra, irrespective of the amount of GO, suggests that this emission does not originate from GO, but most likely arises from DMF degradation by-products. Solvothermal treatment of DMF alone *i.e.*, in absence of GO and H_2_O_2_, results in a pale-yellow liquid solution (inset of Fig. 3c) with a single blue emission peak at 440 nm (Fig. 3c). Although DMF itself does not exhibit fluorescence, it undergoes degradation at high temperatures, likely resulting in the formation of luminescent molecular species. The UV-vis spectrum of this solution (ESI, Fig. S7†) shows peaks at 270 and 340 nm, typically assigned to π–π* and n–π* transitions of C=C and C = X bonds, respectively. Hence, during the solvothermal treatment of GO in DMF, a dual process takes place, involving the formation of green luminescent N-GQDs and the simultaneous decomposition of DMF to generate blue fluorescent by-products, which are not observed in the presence of H_2_O_2_. This study thus establishes a clear relationship between the photoluminescence centres and the liquid reaction products obtained when using the partial reaction pathways. It also highlights the important role of H_2_O_2_ in suppressing fluorescent by-products caused by the self-reaction of DMF.

**Fig. 4:**
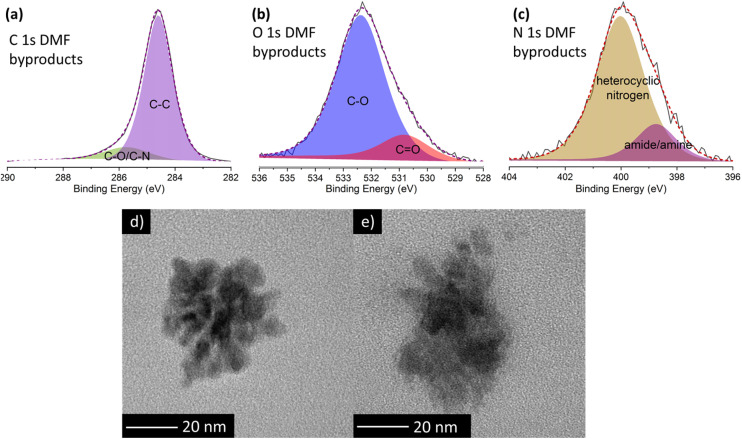
High resolution XPS spectra of (a) C 1s, (b) O 1s and (c) N 1s orbitals of the DMF by-products formed during the treatment of DMF alone. TEM images of the DMF by-products responsible of the blue fluorescence obtained in the reaction of (d) DMF solely and (e) DMF and GO.

To shed more light on the nature of these luminescent nanoparticles derived from DMF, XPS measurements were performed. The XPS survey spectrum (ESI, Fig. S8 and Table S4†) reveals that these by-products mainly consist of carbon (95.3 at%), with minor amounts of oxygen (3.4 at%) and nitrogen (1.3 at%). Deconvolution of the high-resolution C 1s XPS spectrum (Fig. 4a; and ESI, Table S5†) unveils two components: a dominant contribution at 284.6 eV attributed to carbon–carbon bonds (87 at%), and a peak at 285.7 eV corresponding to carbon–oxygen and carbon–nitrogen bonds (13 at%). In the high resolution O 1s spectrum (Fig. 4b), the primary peak at 532.4 eV is attributed to the C–O single bond (83.6 at%), while the peak at 530.8 eV is related to the C=O double bond (16.4 at%). Similarly, in the high-resolution N 1s spectrum (Fig. 4c), the main peak at 400.0 eV corresponds to heterocyclic nitrogen (82.1 at%), while the lower peak at 398.7 eV is ascribed to amide/amine groups (17.9 at%). Notably, the absence of graphitic nitrogen agrees with an emission in the blue region.^28^

TEM images of DMF by-products (Fig. 4d) formed during the self-reaction of DMF show well-defined, globular nanoparticle aggregates lacking any signature of a graphitic structure, which likely explains the blue fluorescent emission. Conversely, in the presence of GO, larger and non-defined formations are identified (Fig. 4e). This difference confirms that GO influences the formation of DMF by-products, hindering the formation of the aforementioned well-defined nanoparticles.

**Fig. 5:**
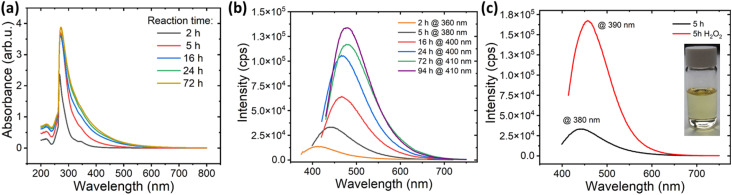
Influence of reaction time on DMF by-products. (a) UV-vis spectra of the solutions obtained from DMF treatment at increasing reaction times. (b) Fluorescence spectra of the blue emission band taken at highest intensity as a function of reaction time for DMF treatment alone. (c) Comparison of the emission spectra of highest intensity after 5 hours reaction time for DMF treatment alone (black curve) and product obtained by adding H_2_O_2_ to DMF (red curve).

To understand the impact of blue-emitting DMF by-products on the final fluorescence, we investigated their luminescent properties as a function of reaction time. Specifically, 10 mL of DMF were treated at 200 °C in an autoclave at increasing reaction times. The resulting solutions show a range of colours from very pale yellow after 2 h of reaction to orange after 94 h (ESI, Fig. S9†). The acquired UV-vis spectra (Fig. 5a) exhibit a main absorption band at 270 nm with a shoulder at 340 nm. The 270 nm band corresponds to the π–π* transition of aromatic sp^2^ domains. Interestingly, the absorbance of the shoulder at 340 nm, related to n–π*transitions involving nitrogen and oxygen functional groups, increases with the reaction time. These features most likely indicate the formation of a larger number of fluorescent species containing nitrogen and/or oxygen at longer reaction times. Moreover, the materials produced for all reaction times exhibit blue emission bands that depend on the excitation wavelength (ESI, Fig. S10†). Remarkably, a ten-fold increase of the maximum emission intensities is detected between 2 h to 94 h (Fig. 5b), emphasising a significant enhancement in the number of fluorescent species as the reaction progresses. Furthermore, the observed redshift from 415 nm to 480 nm with increased reaction times aligns with the formation of larger compounds or the aggregation of the produced species due to their higher concentration.^35,44^ The redshift effect waned in significance beyond 16 h, indicating a stabilization of the maximum emission below that of the N-GQDs. Overall, the intensification of colour and the evolution of UV-vis spectra both provide strong evidence for the formation of a higher number of fluorescent species at long reaction times.

While the thermal decomposition of DMF and other amides at high temperatures is well known phenomenon in organic chemistry, its precise reaction pathways yet remain unclear. Previous studies in biochemistry have reported the self-reaction of formamide, leading to the formation of hydrogen cyanide and various organic compounds, including amino acids, aromatic poly- and heterocyclic molecules, and polymeric compounds consisting of amide or imine chains.^45–50^ Notably, many of these products exhibit photoluminescent properties, probably accounting for the observed blue fluorescence during DMF decomposition in our work. Similar behaviour has been reported for NMP under mild conditions. Its self-reaction, probably through a radical mechanism, results in the formation of fluorescent nanoparticles.^51^ In analogy to our findings with DMF, studies on NMP report on a change in colour of the solution towards a yellow tone and a progressive enhancement of the fluorescence intensity with increasing reaction time, indicating a correlation between reaction time and fluorescence intensity.^52^ To investigate whether the decomposition of DMF follows a radical mechanism and to elucidate the role of H_2_O_2_ as a radical source, we thermally treated 9 mL of DMF at 200 °C during 5 hours in the presence of 1 mL of H_2_O_2_. The resulting solution shows a more intense yellow tone compared to the self-reaction of DMF. While emission spectra are similar (ESI, Fig. S10f†), the addition of H_2_O_2_ leads to a remarkable four-fold increase in emission intensity and a 15 nm redshift compared to DMF alone (Fig. 5c). This fluorescence behaviour, is analogous to the effect of increasing reaction time, and equally indicates the formation of an increased number of fluorescent species derived from DMF. Thus, the addition of H_2_O_2_ as a radical source improves the self-reaction of DMF, which is consistent with a radical degradation mechanism. This suggests that radical species derived from DMF combine to form fluorescent compounds. As the number of radicals increases, either through longer reaction times or presence of a radical source, more blue emitting species are formed, leading to higher overall fluorescence intensity. These results clearly demonstrate that the species formed by the radical self-reaction of DMF are the ones which are responsible for the blue fluorescence observed upon the solvothermal treatment of GO in DMF in the absence of H_2_O_2_.

**Fig. 6:**
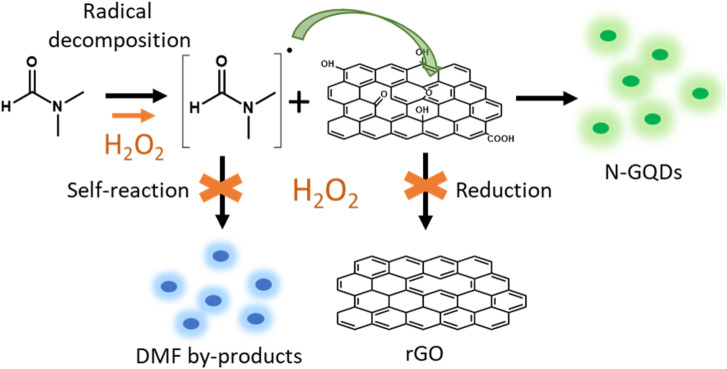
Proposed mechanism for the treatment of GO in DMF in presence of H_2_O_2_. DMF radically decomposes to form reactive nitrogen-containing radicals further reacting with the oxygen functional groups of GO to produce green emitting N-GQDs. H_2_O_2_, acting as a radical source, enhances the reactivity of DMF, avoids the formation of blue emitting by-products from DMF, prevents the reduction of GO, and promotes the formation of N-GQDs.

By contrast, the solvothermal combination of GO, DMF, and H_2_O_2_ eliminates the blue emission entirely. Only the green emission characteristic of N-GQDs remains detectable (Fig. 1a). This suggests that the presence of H_2_O_2_ during the GO solvothermal treatment prevents the formation of fluorescent by-products from DMF. Based on these observations, we propose a mechanism (Fig. 6) in which the first step involves the degradation of DMF and the generation of very reactive nitrogenated radical species. These radicals react with functional groups on GO, mainly C–O groups (epoxy, alcohol), to form N-GQDs by breaking the GO sheets and incorporating nitrogen atoms into the graphenic structure. Thus, the addition of H_2_O_2_ plays a multifaceted role in the synthesis of N-GQDs, performing three key functions. First, H_2_O_2_ acts as a radical source, enhancing the reactivity of DMF and promoting the generation of nitrogenated radicals that readily react with GO. Second, H_2_O_2_ suppresses side reactions inhibiting the self-reaction of the DMF radical species and preventing the formation of unwanted blue fluorescent by-products. Last, H_2_O_2_ optimizes oxidation process, diminishing the reduction effect of DMF on GO and ensuring reaction pathways towards N-GQD formation without interruption. This triple role of H_2_O_2_ promotes the selective formation of green-emitting N-GQDs by inhibiting undesirable side-reactions.

## 4 Conclusions

This study presents a straightforward and effective method for the synthesis of genuinely green fluorescent N-GQDs. The key innovation lies in the addition of H_2_O_2_ to the solvothermal reaction involving GO and DMF. This synergistic combination not only promotes the formation of the desired green fluorescent N-GQDs but also suppresses the generation of unwanted blue-emitting byproducts. Our investigations reveal that H_2_O_2_ plays a crucial threefold role in the reaction mechanism: first, it acts as a radical source facilitating the degradation of DMF and the generation of essential nitrogen-containing radicals. Second, as an oxidant, H_2_O_2_ prevents the thermal reduction of graphene oxide, and hence, ensures its continued reaction with the DMF-derived radicals, ultimately leading to N-GQD formation. Third, H_2_O_2_ inhibits the formation of blue-emitting DMF-derived by-products, thereby enabling the targeted synthesis of N-GQDs exclusively exhibiting green fluorescence. In conclusion, this work demonstrates that incorporating H_2_O_2_ in the solvothermal treatment of GO with DMF offers a promising approach for the controlled synthesis of N-GQDs with well-defined long-wavelengths emission properties. The elucidation of the reaction mechanism and the multifaceted roles of H_2_O_2_ opens new possibilities for the rational design and optimization of N-GQD synthesis protocols.

## Data availability

The data supporting this article have been included as part of the ESI.†

## Author contributions

C. C. and A. G. G. carried out the experimental work. Analysis of experimental data was performed by C. C., A. G. G. and A. M. B. A. M. B. and W. K. M. conceptualized the work. Supervision was shared between A. M. B. and W. K. M. The manuscript was drafted by C. C. and A. G. G. and finalized by all authors. Funding was acquired by A. M. B and W. K. M. Data curation is ensured by A. M. B. and W. K. M.

## Conflicts of interest

There are no conflicts to declare.

## Supplementary Material

SC-OLF-D4SC07896A-s001
