# Supplementary material for: Multifaceted role of H2O2 in the solvothermal synthesis of green-emitting nitrogen-doped graphene quantum dots
Source: Chem Sci. 2025 Jan 28;16(8):3662–70. doi: 10.1039/d4sc07896a (PMC11773602; doi:10.1039/d4sc07896a)
Supplement: SC-016-D4SC07896A-s001 [file SC-016-D4SC07896A-s001.pdf]

Supplementary Information (SI) for Chemical Science.
This journal is © The Royal Society of Chemistry 2025

# Supplementary Information

# Multifaceted role of $H_2O_2$ in the solvothermal synthesis of green-emitting nitrogen-doped graphene quantum dots

Clara Carrera,[a] Alejandro Galán-González,[a] Wolfgang K. Maser,[a] and Ana M. Benito*[a]

[a] Instituto de Carboquímica (ICB-CSIC), C/Miguel Luesma Castán 4, E-50018 Zaragoza (Spain)

*E-mail: abenito@icb.csic.es

## Index

S1. Synthesis of graphene oxide (GO)

S2. Transmission electron microscopy (TEM) of GO

S3. Transmission electron microscopy (TEM) of N-GQDs

S4. Thermogravimetric analysis (TGA), Raman and FT-IR spectra of GO and N-GQDs

S5. X-ray photoemission spectroscopy (XPS) of GO and N-GQDs

S6. Powder X-ray diffraction (XRD) of GO and filtered reaction products

S7. Emission spectra from products of GO reacted in DMF in different amounts

S8. Characterization of DMF by-products: UV-vis spectra, XPS, photos of solutions, emission spectra as a function of reaction time

## S1. Synthesis of graphene oxide (GO)

Graphite oxide was prepared according to a modified Hummers method.[1,2] Specifically, graphite flakes (5 g) were dispersed into a mixture of $H_2SO_4$ (170 mL) and $NaNO_3$ (3.75 g) by stirring for 30 min on an ice bath. Afterwards, $KMnO_4$ (25 g) was slowly added and the reaction was cooled at 0 °C during 30 min. Thereafter, the mixture was warmed up to 35–40 °C and kept stirring overnight. The slowly addition of deionized water (250 mL) and $H_2O_2$ (20 mL, 30%) solution concluded the reaction. Subsequently, the dispersion was filtered and the solid is repeatedly washed firstly with HCl: $H_2O$ (400 mL, 1:10 v/v) and secondly with deionized water up to neutral pH. The product was dried at room temperature to obtain graphite oxide powder. Subsequent ultrasonication in the employed reaction solvent (DMF, $H_2O$, $H_2O_2$) leads to well exfoliated graphene oxide sheets.

## References


1 W. S. Hummers, R. E. Offeman, *J. Am. Chem. Soc.* 1958, ***80***, 1339.

2 E. Colom, J. Hernández-Ferrer, A. Galán-González, A. Ansón-Casaos, M. Navarro-Rodríguez, E. Palacios-Lidón, J. Colchero, J. Padilla, A. Urbina, R. Arenal, A. M. Benito, W. K. Maser, *Chem. Mater.* 2023, ***35***, 3522.

## S2. Transmission electron microscopy (TEM) of graphene oxide (GO)

The solid precipitate obtained in the reaction of GO with $H_2O_2$ in DMF was dispersed in ethanol and a diluted fraction drop casted onto copper TEM grids coated with a lacey carbon film. The prepared sample was allowed to dry before introduced into the TEM microscope. TEM micrographs of pristine GO (Fig. S1 (a) and (b)) show individual sheets having lateral sizes of a few micrometers. Their high transparency reveals sheets consisting of one or only very few layers. The sheet edges are smooth but also exhibit sites with contrast in thickness indicating the folding of the GO sheets (red box). Conversely, TEM images of the non-soluble black solid fraction obtained after the solvothermal process, (Figures S1 (c) and S1 (d)), reveal the presence of GO sheets having suffered important modifications under the harsh synthesis conditions. Firstly, the observed flakes appear quite dense, revealing an important restacking of the parent GO sheets. Second, the edges of the restacked individual sheets appear quite rough. These distinct features are indicative of chemical attacks, favouring the cleavage of GO sheets to successfully generate small sized N-GQDs out from its parent structure. While these are present in the supernatant fraction, the remaining chemically attacked parent GO sheets restack, most likely due to significant changes in their surface chemistry, thus affording the solid reaction precipitate consisting of restacked and partially reduced GO sheets, in agreement with the X-ray diffraction study of these materials (see S6).

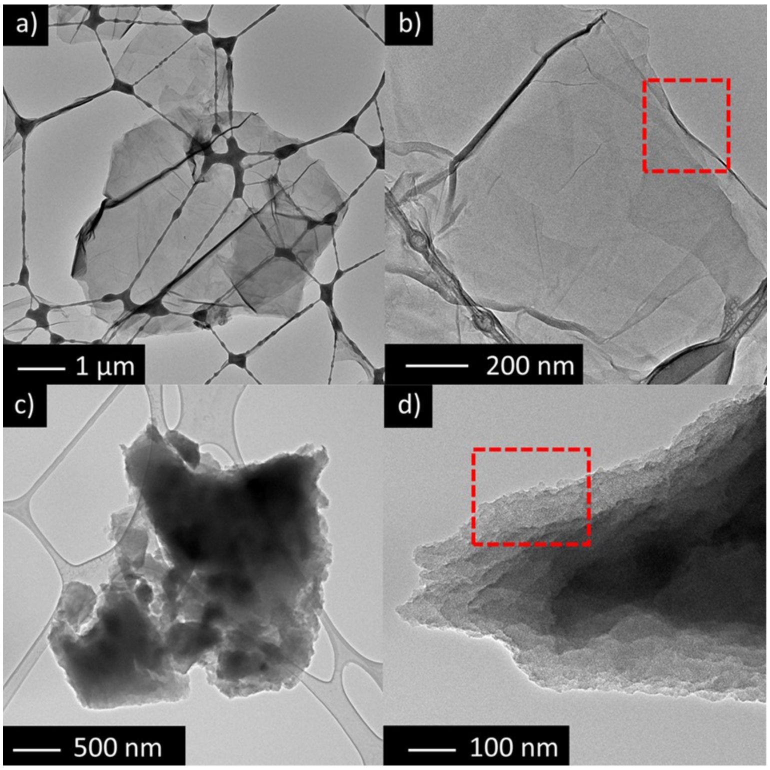


**Figure S1.** TEM images of (a,b) pristine GO and (c,d) the precipitated solid product fraction obtained in the reaction of GO with $H_2O_2$ in DMF. Red squares highlight the edge morphology.

**S3. Transmission electron microscopy (TEM) of N-GQDs**

The filtered and diluted N-GQDs dispersion was drop casted copper TEM grids coated with a lacey carbon film. The prepared sample was allowed to dry before introduced into the TEM microscope. TEM inspection (Fig. S2a) reveal the presence of aggregated N-GQDs. The size of the aggregates is about 16 nm (manuscript Fig. 1.a). A magnification of aggregated N-GQDs is shown in Figure S2b. Crystalline lattice fringes in different parts of the aggregates are identified exhibiting a size of about 0.22 nm (Figure S2c). Being in agreement with the typical in-plane (100) lattice distance of a graphene sheet, this observation underscores the graphenic structure of the N-GQDs and thus the direct relationship with the parent GO sheets. Although the overlapping aggregate structures exhibit fringes belonging to different N-GQDs, the crystallite domain sizes of individual N-GQDs estimated from these images most likely does not extend beyond 10 nm.

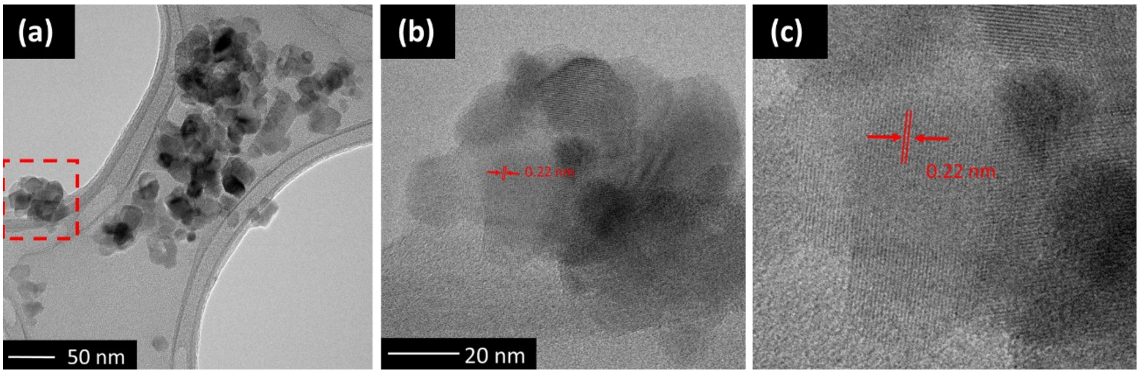


**Figure S2.** TEM image N-GQDs. (a) low magnification of aggregated N-GQDs; (b) magnification of aggregated N-GQDs corresponding to the red-marked area in (a); (c) an amplified zoom of (b), clearly showing lattice fringes within the aggregated N-GQDs.

## S4. TGA, Raman, and FT-IR results of GO and N-GQDs

A comparative study on N-GQDs and the parent GO by thermogravimetric analysis (TGA), Fourier -transform infrared (FT-IR) spectroscopy and Raman spectroscopy was performed. Results are shown in Figure S3.

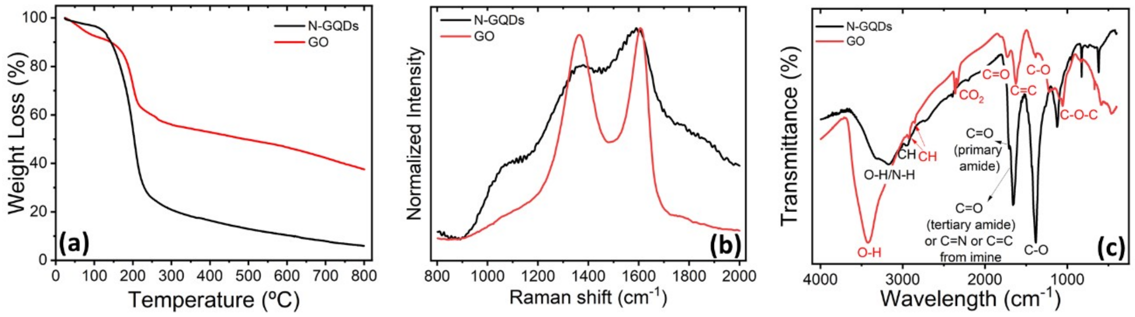


**Figure S3.** Comparison of N-GQDs and GO. (a) Thermogravimetric analyses (TGA), (b) Raman spectra, and (c) Fourier-transform infrared spectra (FT-IR).

TGA (Figure S3a) was performed under nitrogen to evaluate the thermal stability and surface composition of GO and N-GQDs. GO exhibits a two-step weight loss profile. An initial 10 wt. % loss below 115 °C is attributed to the desorption of physisorbed water. A more substantial 30 wt. % loss around 200°C corresponds to the removal of chemisorbed water and associated less stable oxygen functional groups. From 230°C to 800°C, a gradual weight loss occurred due to the decomposition of more stable oxygen functional groups.[3] The total weight loss at 800°C was 60 wt. %. N-GCDs show a 3 wt. % below 115 °C, corresponding to the removal of physisorbed water. Furthermore, N-GCDs reveal a rapid and extensive weight loss of approximately 75 wt. % at 200°C, due to the removal of oxygen functional groups. At 800 °C, only 6 wt. % of the original material remains. This accelerated decomposition highlights the lower thermal stability of N-GCDs compared to GO, as consequence of their increased surface area, as well as their higher density of functional groups per unit mass compared to GO.

Figure S3b shows the normalized Raman spectra of GO and N-GQDs. The characteristic G and D bands at 1366 $cm^{-1}$ and 1605 $cm^{-1}$ are clearly observed in the Raman spectrum of GO, indicative for both $sp^3$ carbon and $sp^2$ hybridized carbon domains, respectively.[4] The comparable intensities of both bands are indicative of the highly defective nature, corresponding to its heavily oxidized structure of the starting GO material, typical for what is observed for well-exfoliated GO sheets. N-GQDs equally show the corresponding G and D bands at the same position, underscoring their direct relationship to the parent GO structure. Although the apparent change in the intensity ratio of the D and G band their increased bandwidth for N-GQDs is probably influenced by the strong fluorescent background it confirms the successful

transformation of the non-fluorescent parent GO sheets into fluorescent N-GQDs of smaller crystallite domain sizes.

FT-IR spectra of N-GQDs and GO (Fig. S3c) with indication of corresponding vibrational modes reveal the close relationship between N-GQDs and the parent GO, but also clearly disclose the presence of nitrogen-containing functional groups in N-GQDs. In the case of GO, the observed signals can be associated to the carbon structure (C=C at 1625 $cm^{-1}$ and C-H at 2950 $cm^{-1}$) and oxygen functional groups (C-O at 1050 $cm^{-1}$, C-OH at 1390, $cm^{-1}$ C=O at 1725 $cm^{-1}$ and O-H at 3425 $cm^{-1}$). It is important to note that water scissor mode (deformation vibration) can overlap with the C=C stretching vibration peak at 1625 $cm^{-1}$, contributing to the observed signal. By comparison, the spectrum of N-GQDs exhibits a combination of modes for conjugated C=C (together with water scissor), C=N and the C=O (shoulder) in the region of 1660 $cm^{-1}$. Additionally, the presence of nitrogen functional groups is also confirmed by new signals in the 3400-3000 $cm^{-1}$ region (N-H) and a peak at 1380 $cm^{-1}$, indicative of strong C-N vibration modes corresponding to amides. Thus, the FTIR spectrum suggests that nitrogen is incorporated into the structure in many different forms such as amines, amides and N-heterocyclic groups. These results align well with XPS observations from the high resolution N1s spectrum, further underlining the incorporation of nitrogen in various forms such as amines, amides, N-heterocyclic groups (pyrrolic, pyridinic) or graphitic nitrogen.

In summary, the results from TGA, FT-IR and Raman consistently confirm the transformation of the heavily oxidized and well exfoliated parent GO sheets into graphene dots with nitrogen functionalities incorporated in their structure upon the solvothermal synthesis process, thus resulting in fluorescent N-QGDs. This is in full agreement with all other specific findings of this work.

**References:**


3 J.D. Núñez, A.M. Benito, S. Rouziére, P. Launois, R. Arenal, P.M. Ajayan, W.K. Maser, *Chem.Sci*. 2017, **8**, 4987-4995.

4 A.C. Ferrari, J. Robertson, *Phys. Rev*. B 2000, **61**, 14095–107.

## S5. X-ray photoemission spectra (XPS) of GO and N-GQDs

### Survey spectra

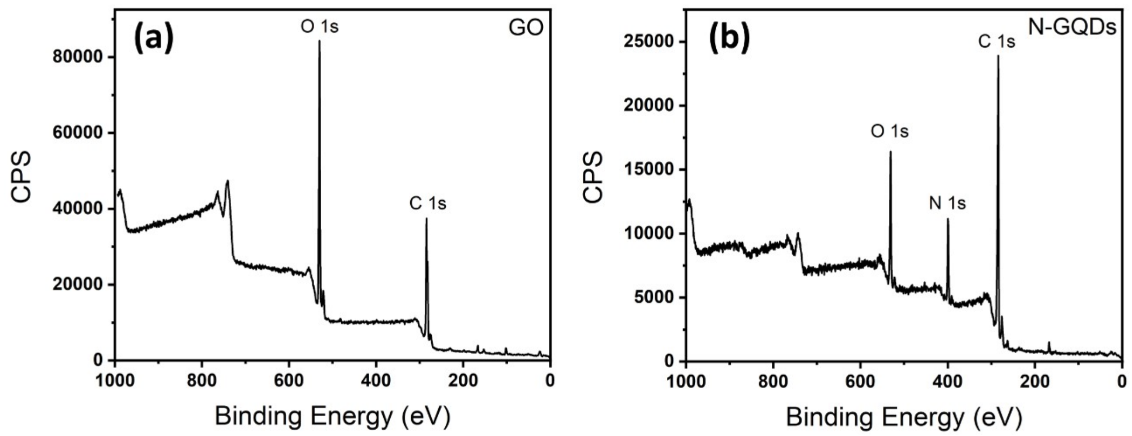


**Figure S4.** XPS survey spectra of (a) GO and (b) N-GQDs.

**Table S1.** Analysis of the survey spectra of GO and N-GQDs.

| Sample | Carbon (at. %) | Oxygen (at. %) | Nitrogen (at. %) |
|---|---|---|---|
| GO | 65 | 35 | -- |
| N-GQDs | 76 | 13 | 11 |

### Deconvolution results of the high resolution XPS spectra of N 1s, C 1s, and O 1s orbitals of GO and N-GQDs

**Table S2.** Deconvolution of C 1s, O 1s and N 1s orbitals from high resolution XPS spectra of GO and N-GQDs, indicating the position (eV) and area contribution (at. %).

| C 1s | C $sp^2$ | | C $sp^3$ | | CO/CN | | COO | |
|---|---|---|---|---|---|---|---|---|
| | (eV) | (at. %) | (eV) | (at. %) | (eV) | (at. %) | (eV) | (at. %) |
| GO | 284.6 | 13.1 | 285.4 | 24.9 | 287.4 | 54.2 | 289.2 | 7.8 |
| N-GQDs | 284.5 | 16.4 | 285.1 | 29.6 | 286.0 | 45.1 | 288.2 | 8.9 |

| O 1s | C=O | | C-O | |
|---|---|---|---|---|
| | (eV) | (at. %) | (eV) | (at. %) |
| GO | 531.6 | 47.2 | 532.4 | 52.8 |
| N-GQDs | 531.3 | 54.3 | 532.6 | 45.7 |

| N 1s | Amides /amines | | Heterocyclic nitrogen | | Graphitic nitrogen | |
|---|---|---|---|---|---|---|
| | (eV) | (at. %) | (eV) | (at. %) | (eV) | (at. %) |
| N-GQDs | 399.2 | 22.6 | 400.2 | 74.8 | 402.4 | 2.6 |

**S6. Powder X-ray diffraction (XRD) of GO and precipitated reaction products**

XRD diffraction patterns were collected from powder samples of the filtered products. (Figure S5)The XRD pattern of pristine GO shows the characteristic (001) diffraction peak at 2θ □□10°, corresponding to an interlayer spacing of about□0.88 nm, indicating the existence of oxygen functional groups (OFGs) on the basal plane of GO, as well as the presence of physisorbed and chemisorbed water.[3-5] This diffractogram thus indicates the presence of well exfoliated hydrated GO sheets randomly restacked in the solid state.[3] In the filtered reaction products obtained from both syntheses (DMF and DMF + $H_2O_2$), only a very broad peak at 2θ □□25° is now observed, corresponding to an significantly decreased interlayer spacing of 0.36 nm. The lower distance of the restacked GO sheets reveals a partial reduction of GO due to the loss of OFGs (and associated water) on the basal sheet planes of GO,[3-5] while its broadness indicates the amorphous nature of the precipitated products. Notably, the relative intensity of this diffraction peak was higher in the absence of $H_2O_2$, suggesting a larger degree of OFG removal under these conditions.

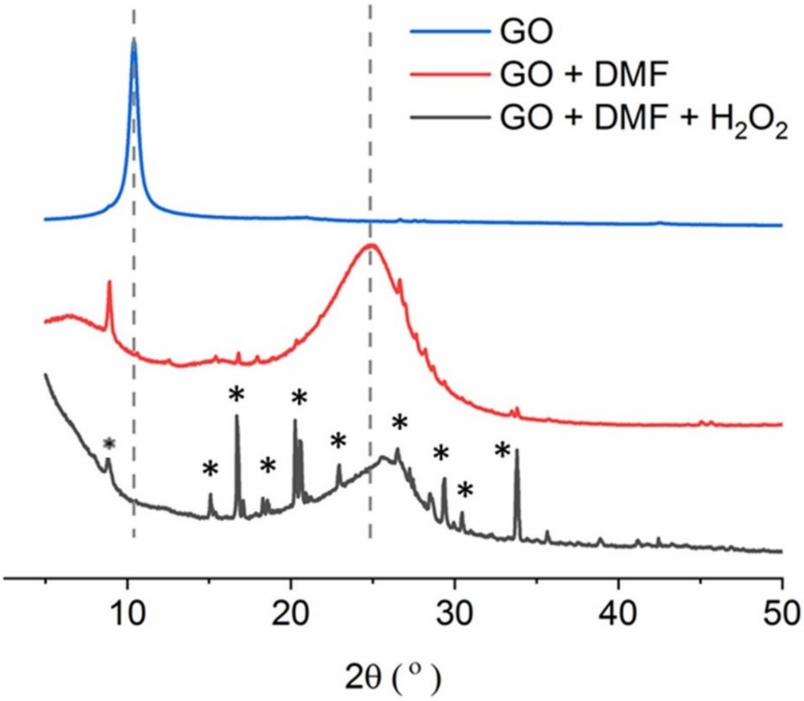


**Figure S5**. XRD pattern of pristine GO, and the solid precipitates obtained after reaction with DMF alone or $H_2O_2$ and DMF jointly. The peaks marked with * correspond to the signals of the filter used for the separation of the solid fraction and can be observed in both samples, GO + DMF + $H_2O_2$ (N-QGDs) and GO + DMF, respectively.

Crystallite sizes L were determined using the Scherrer equation[6]

$$L = K \cdot \lambda / B \cdot \cos\theta,$$

where B and θ correspond to the FWHM and position θ of a diffraction peak, respectively; K is a dimensionless constant, typically set to 0.9 and λ the wavelength delivered from the anode of the XRD apparatus, in our case a copper anode $CuK_{\alpha}$ with a $\lambda = 0.15418$ nm.

Crystallite sizes L were calculated from the position and FWHM of the (001) peak of GO and the (001) peak of the GO + DMA and GO + DMF + $H_2O_2$ (N-GQD) sample, resulting in the following values (Table S1)

**Table S3:** Peak position, FWHM and crystallite size for GO and solid precipitated products from the reaction with of GO with DMF and GO with DMF and $H_2O_2$.

| **Sample** | **Peak position 2θ (°)** | **FWHM (Radian)** | **L (nm)** |
|---|---|---|---|
| **GO** | 10.3986 | 0.01066065 | **13.07** |
| **GO + DMF** | 24.8442 | 0.08445404 | **1.68** |
| **GO + DMF + $H_2O_2$** | 25.7340 | 0.08710519 | **1.63** |

The small crystallite sizes of the solid precipitated products reflect the chemical modification of the GO sheets under the harsh synthesis conditions, as discussed in TEM section S1.

**References:**


3 J.D. Núñez, A.M. Benito, S. Rouziére, P. Launois, R. Arenal, P.M. Ajayan, W.K. Maser, *Chem.Sci*. 2017, **8**, 4987-4995.

4 S. Rouzière, P. Launois, A.M. Benito, W.K. Maser, E. Paineau, *Carbon* 2018, **137**, 379-383.

5 S. Rouzière, J.D. Núñez, E. Paineau, A.M. Benito, W.K. Maser, P. Launois, *J. Appl. Cryst*. 2017, **50**, 876-884.

6 D.J. Lim, Nigel A. Marks, M.R. Rowles, *Carbon* 2020, **162**, 475-480.

**S7. Emission spectra from products of GO reacted in DMF in different amounts**

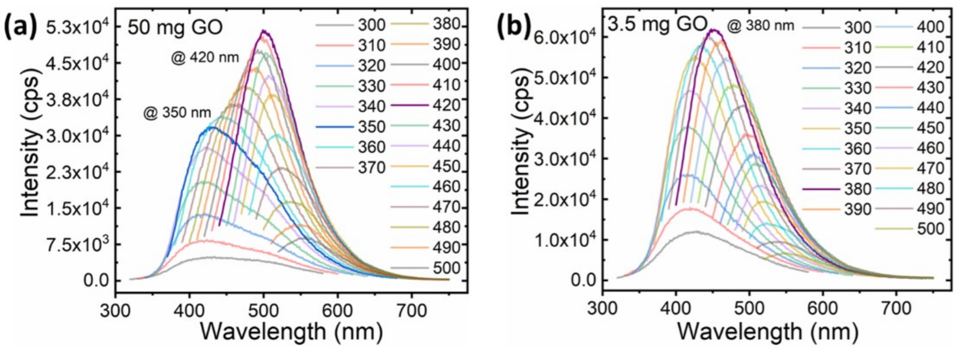


**Figure S6.** Emission spectra of the treatment in DMF of (a) 50 mg and (b) 3.5 mg of GO. The excitation used increases by 10 nm from 300 nm to 500 nm (colour code for the excitation wavelengths is included). The excitation wavelengths corresponding to the emission maxima are indicated. The emission maxima are obtained in the green region when using 50 mg of GO (a) and in the blue region when using 3.5 mg of GO.

## S8. Characterization of DMF by-products

### UV-vis spectra

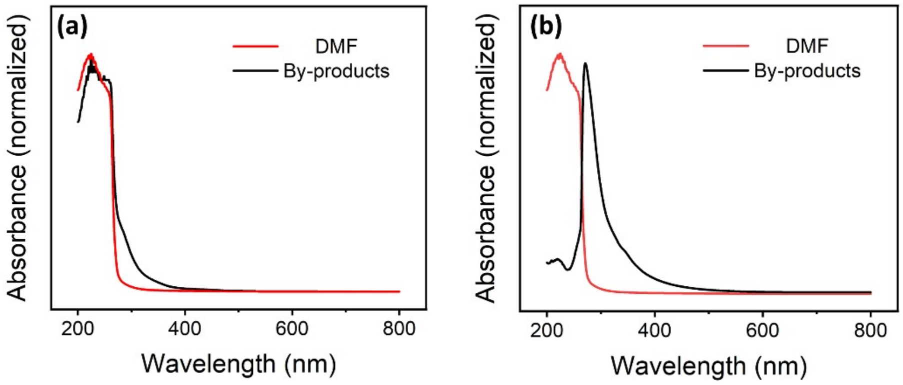


**Figure S7**. Comparison of the normalized UV-vis spectra of DMF and the by-products obtained by the solvent degradation under the standard conditions. (a) Pristine spectra and (b) Spectra of by-products with subtracted DMF contribution.

### XPS survey spectrum

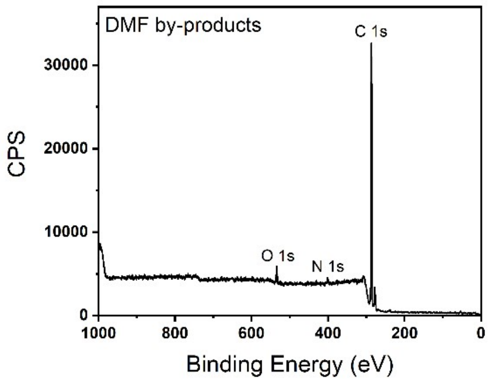


**Figure S8.** XPS survey spectrum of DMF by-products

**Table S4.** Analysis of the survey spectrum of DMF by-products.

| Sample | Carbon (at. %) | Oxygen (at. %) | Nitrogen (at. %) |
|---|---|---|---|
| **DMF by-products** | 95.3 | 3.4 | 1.3 |

### Deconvolution results of the high resolution XPS spectra of C 1s, O 1s and N 1s orbitals of DMF by-products

**Table S5.** Position (eV) and area (at. %) of the deconvoluted high resolution XPS spectra of C 1s, O 1s and N 1s orbitals from DMF by-products.

| C1 s | | | | O 1s | | | | N 1s | | | |
|---|---|---|---|---|---|---|---|---|---|---|---|
| C-C | | C-O/ C-N | | C=O | | C-O | | Amides/ Amines | | Heterocyclic nitrogen | |
| (eV) | (at. %) | (eV) | (at. %) | (eV) | (at. %) | (eV) | (at. %) | (eV) | (at. %) | (eV) | (at. %) |
| 284.6 | 87.0 | 532.4 | 13.0 | 530.8 | 16.4 | 285.7 | 83.6 | 398.7 | 17.9 | 400.0 | 82.1 |

**Photos of solutions of DMF by-products as a function of reaction time**

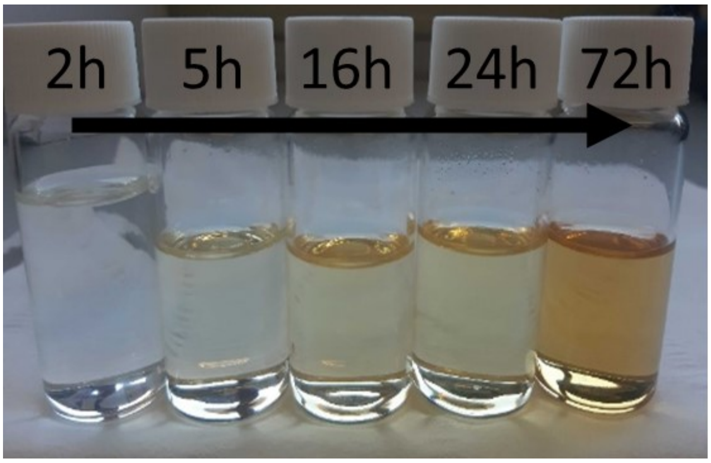


**Figure S9**. Colour evolution of the reaction of DMF at 200 $^oC$ for 2, 5, 16, 24 and 72 hours, showing darkening of the dispersion as the time increases, being attributed to an increment of the number of nanoparticles with time.

**Emission spectra of solutions of DMF by-products as a function of reaction time**

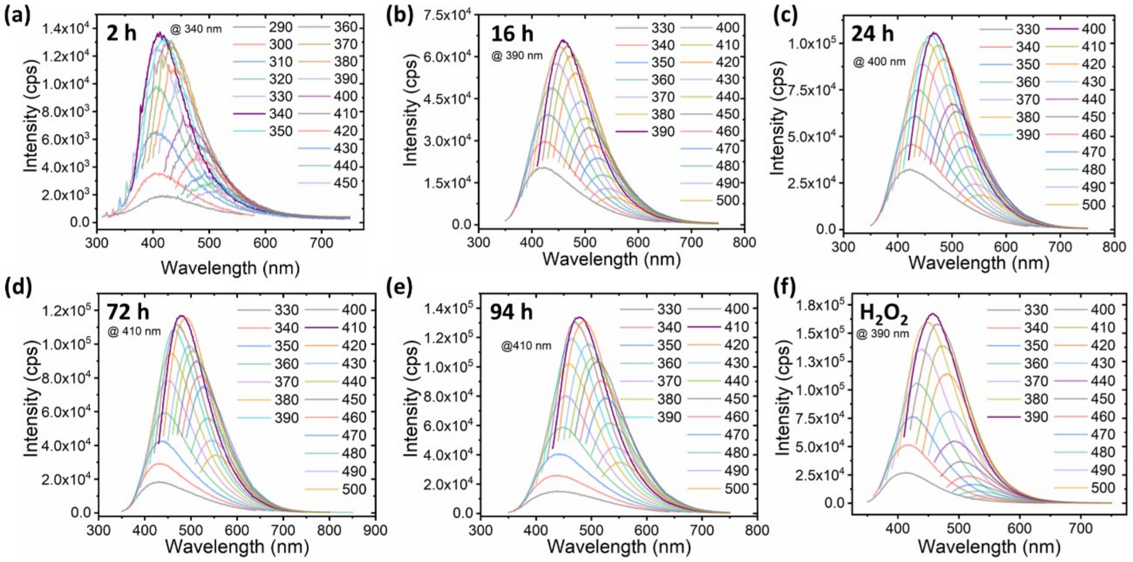


**Figure S10.** Emission spectra of the treatment of DMF at 200$^oC$ during (a) 2 h, (b) 16 h, (c) 24 h, (d) 72 h or (e) 94 h and (f) the treatment with $H_2O_2$ for 5 h. The colour codes for the excitation wavelengths is included in the respective figures. The excitation wavelengths corresponding to the emission maxima are indicated, revealing that the maximum emission intensity is achieved when exciting in the region of 390 – 410 nm.
